# Leveraging Genetics to Advance Type 2 Diabetes Prevention

**DOI:** 10.1371/journal.pmed.1002102

**Published:** 2016-07-26

**Authors:** Jose C. Florez

**Affiliations:** 1 Diabetes Unit and Center for Human Genetic Research, Massachusetts General Hospital, Boston, Massachusetts, United States of America; 2 Programs in Metabolism and Medical & Population Genetics, Broad Institute, Cambridge, Massachusetts, United States of America; 3 Department of Medicine, Harvard Medical School, Boston, Massachusetts, United States of America

## Abstract

In this Perspective, Jose Florez discusses how information from genetics and genomics may be able to contribute to prevention of type 2 diabetes and predicting individual responses to behavioral and other interventions.

Type 2 diabetes is a preventable disease. The pandemic that has reached 415 million people worldwide continues to grow, threatens to undermine the global economy, and is taking place on the back of a profound transformation in the modern lifestyle, which involves pervasive detrimental changes in diet and physical activity patterns [1]. Though the disease undoubtedly has a genetic component, the recent explosion in its prevalence is clearly driven by a dynamic environment operating on a relatively static genetic background. But this equation also has a favorable counterpoint: as a deleterious environment has driven diabetes prevalence into double percentage digits in just a few decades, so reverting this trend by the widespread introduction of healthy behavioral patterns should, in turn, reduce disease burden.

Lifestyle intervention strategies have been proven to be effective in randomized controlled trials. The Chinese Da Qing study [2], the Finnish Diabetes Prevention Study [3], the U.S. Diabetes Prevention Program (DPP) [4], and the Indian Diabetes Prevention Program [5] have consistently shown that intensive lifestyle modification is able to prevent, in some, and delay, in others, the incidence of diabetes, despite the high-risk metabolic profile of participants. Lifestyle interventions are successful across the full range of genetic risk (Fig 1) [6,7] and can also reduce cardiovascular mortality if given enough time [8]. However, many of the prediabetic participants continue to march toward diabetes, albeit at a slower pace [9], and deploying a successful lifestyle intervention across the entire population at risk (~52 million in North America) is a massive undertaking.

**Fig 1:**
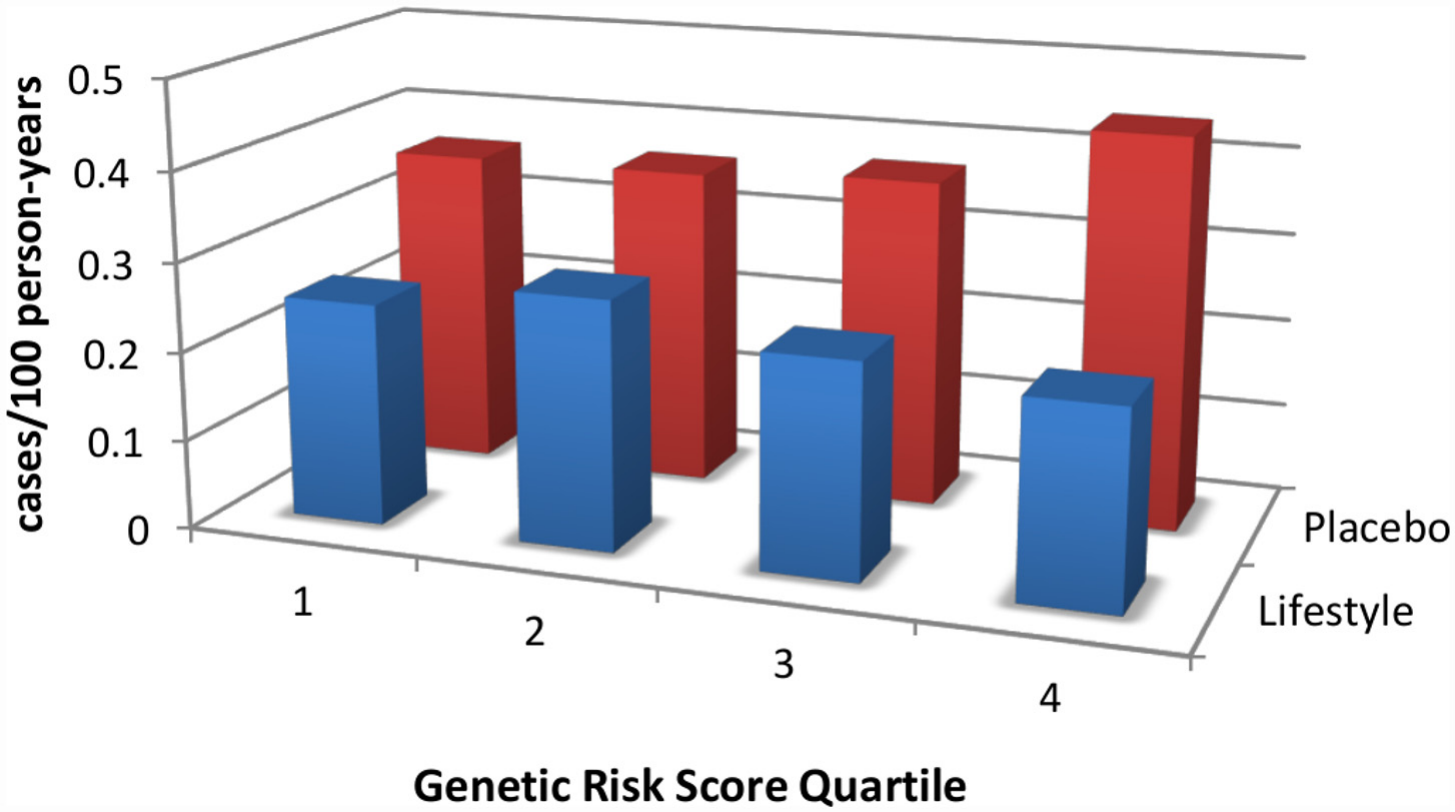
An intensive lifestyle intervention, as deployed in the U.S. Diabetes Prevention Program (DPP), is effective regardless of genetic risk score for type 2 diabetes. DPP participants were stratified by quartile of genetic risk constructed by adding risk alleles from 34 known type 2 diabetes-associated variants. Whereas the genetic risk score predicts diabetes incidence in the placebo arm (red bars), it does not do so in the lifestyle arm (blue bars); indeed, the intervention is highly effective in reducing diabetes incidence even in the quartile with the highest genetic risk. Data are from reference [11].

While logistically challenging, preventive strategies do make financial sense. An economic analysis of participants who adhered to the intervention in the DPP found that, over a ten-year period, intensive, individualized lifestyle modification costs less than US$5,000 per quality-adjusted life year [10], well within the expense range of other health interventions routinely embraced by high-income societies. Furthermore, preventive treatment with metformin was found to be cost-saving for those participants who adhered to treatment in the DPP study. However, these interventions would still involve a substantial investment, and governments are not necessarily in a position to provide such interventions to all their citizens at risk.

## Identifying Individuals at Increased Risk

In a world of finite resources, it may make sense to prioritize those most likely to benefit. Therefore, great interest has emerged in whether nascent technologies can be used to identify individuals at risk of future type 2 diabetes and/or predisposed to experience a favorable response to preventive strategies.

Among all available approaches for more detailed personalized profiling, genetics has led the way for a number of reasons. First, it is now possible to query millions of variants across the human genome in a single experiment with great precision, and to interpret the ensuing results with appropriate statistical rigor. Second, germline genetic variation is fixed in the individual, and thus need be measured only once in the person’s lifetime. Third, DNA is easily accessible and does not require the biopsy of type 2 diabetes-relevant tissues such as beta cells, which would be required for many other biological modalities.

**Fig 2:**
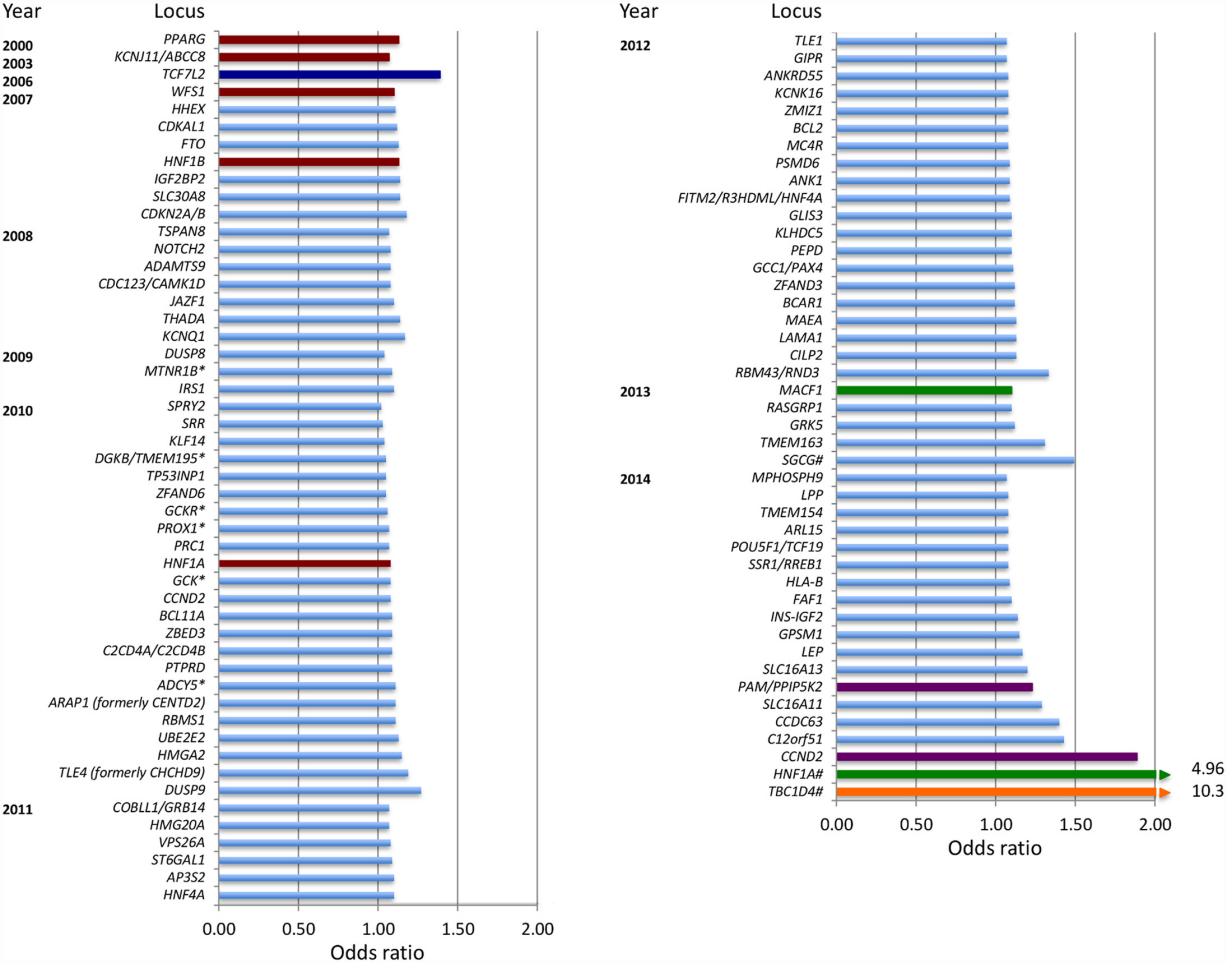
Chronological listing of type 2 diabetes-associated loci, plotted by year of definitive publication and approximate effect size. By convention, the gene name closest to the index variant is given. Loci identified via the candidate gene approach are shown in red, loci identified via agnostic genome-wide association approaches are shown in light blue, loci identified by exome sequencing are shown in green, and loci identified by whole-genome sequencing are shown in purple. *TCF7L2* (shown in dark blue) was discovered by dense fine-mapping under a linkage signal. *TBC1D4* (shown in orange) was identified by exome sequencing of a locus found to be associated with a diabetes-related quantitative trait. Gene names that have asterisks (*) indicate initial discovery by association with quantitative glycemic traits; gene names that have a number sign (#) denote identification in population isolates.

Advances in genotyping and sequencing, as well as plummeting costs and widespread international collaboration, have led to the identification of over 100 genetic variants associated with type 2 diabetes or related glycemic traits [12]. Most of them are common and shared across populations and, with a few exceptions [13,14], tend to have a small effect on risk (odds ratios 1.1–1.4; Fig 2). Combined genetic risk scores composed by the weighted sum of the risk alleles at these loci have been tested for their ability to predict diabetes in individuals, above and beyond the information provided by clinical risk factors. However, the available clinical prediction tools are good, they include variables (such as fasting glucose or body mass index) that already capture genetic information, and the combined effect of these genetic variants is modest; thus, genetic data do not seem to add much to clinical information in type 2 diabetes prediction [15–17]. The addition of metabolomic biomarkers only offers marginal additional benefit [18], and the incremental genetic information only reclassifies a few individuals into more accurate categories of risk. Genotype scores perform slightly better in younger subgroups or those with longer follow-up [15,16].

**Fig 3:**
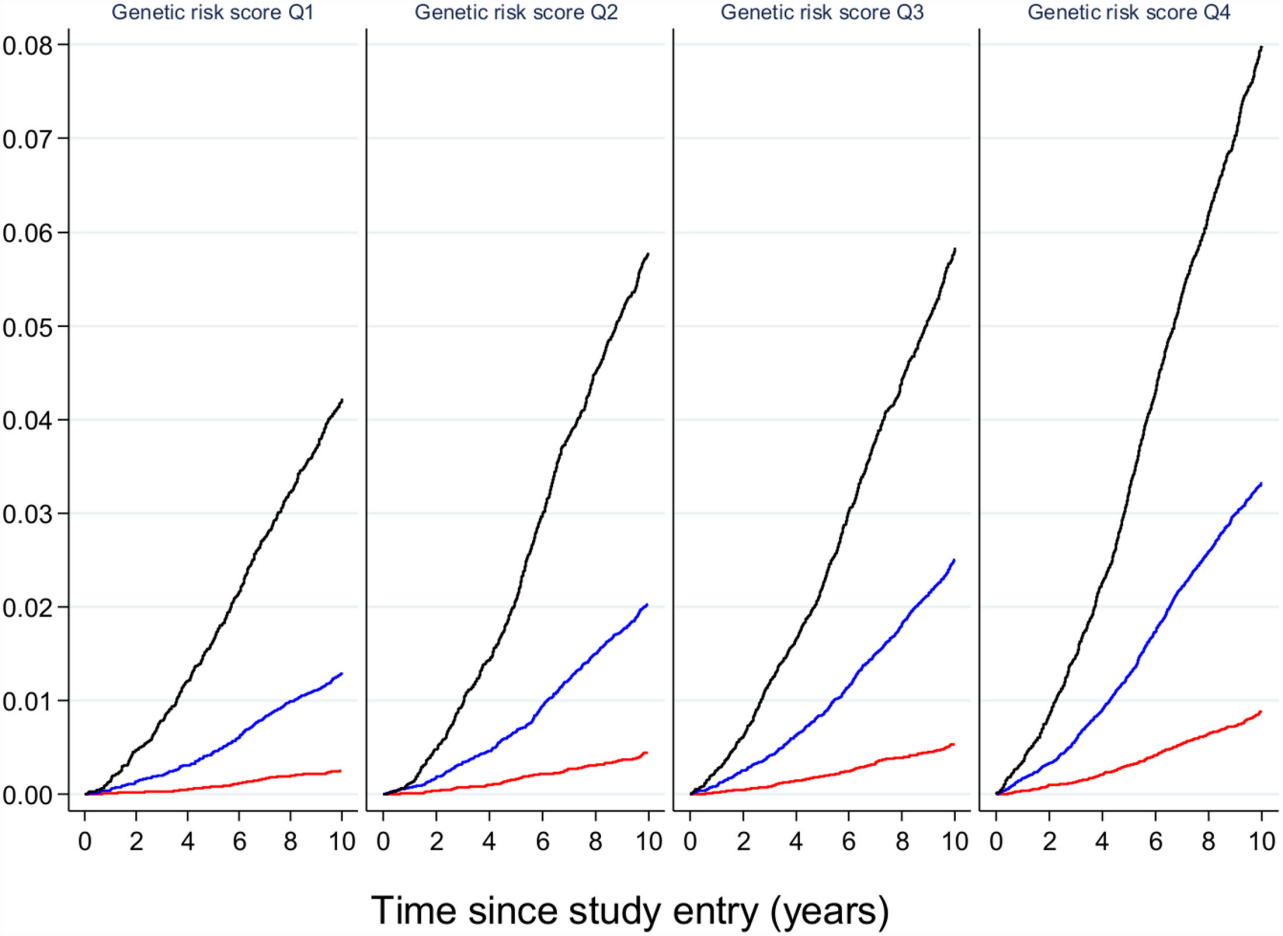
The relative effect of genetic risk and obesity on future type 2 diabetes. Participants in the EPIC-Interact study were stratified by quartiles of genetic risk and by strata of adiposity (obese in black, overweight in blue, and normal weight in red). The likelihood of developing type 2 diabetes is shown, indicating that obese participants with the lowest genetic burden have a higher absolute risk of diabetes than lean or overweight participants with the highest genetic burden. Image is from Langenberg et al. [19].

These results suggest that, if genetic prediction tools composed of common genetic variants were to be deployed to enhance prediction and target subgroups for preventive interventions, they would need to be combined with other risk factors that capture the global risk profile of an individual. As an illustration of this concept, Claudia Langenberg and colleagues used the EPIC-Interact study to demonstrate that, for individualized risk prediction, absolute risk, and not relative risk, is what matters. They stratified participants by their genetic risk scores as well as by their obesity categories (obese, overweight or normal weight). Obese individuals in the lowest genetic risk quartile were much more likely to develop type 2 diabetes than normal-weight individuals in the highest genetic risk quartile (Fig 3), indicating that if only genetics had been used for risk stratification, the individuals at highest risk would not have been targeted for intervention [19].

While this is the case for common genetic variants of small effects shared across populations, a potential exception might be made for variants that have much stronger effects. Explorations that target a lower frequency of the allelic spectrum have led to the identification of loci that are unique to a specific ethnic group but are present at appreciable frequencies in members of that group. For example, whole-exome sequencing in ~4,000 people of Latino descent uncovered the coding variant E508K in the gene *HNF1A*, which increases diabetes risk 5-fold [13]. Though complete loss of function in the transcription factor HNF1A causes maturity-onset diabetes of the young (MODY) type 3, the E508K allele only attenuates transcriptional activity by ~60%; as a result, the clinical phenotype of carriers is indistinguishable from regular type 2 diabetes, which would not alert the clinician to a mutation in a known MODY gene. The variant is carried by ~2% of Mexicans with type 2 diabetes, and, given its large impact on diabetes risk, it may help to target carriers (identified because they have a prediabetic phenotype, or they are relatives of a diabetic carrier, or they have undergone population screening when available) for more intensive surveillance and prevention. Similarly, Ida Moltke and colleagues identified a nonsense variant in the gene *TBC1D4*, which is present in 17% of Inuit individuals in Greenland [14]. Loss of function of its protein product interferes with insulin-stimulated Akt-induced glucose uptake in skeletal muscle, and the variant is thus associated with higher two-hour glucose and a 10-fold increased risk of type 2 diabetes. The clinician caring for this population would do well in using oral glucose tolerance tests to diagnose prediabetes and diabetes, to ensure that the 2-hour glucose measure is considered in diagnostics, and carriers might be better treated with an insulin sensitizer for either diabetes prevention or treatment.

## Identifying Individuals More Likely to Respond to Intervention

While the value of genetic testing in type 2 diabetes prediction is limited, it may have a more useful role in the selection of patients more likely to respond to intervention. Though many of these observations require independent replication, the DPP Research Group has shown that variation in the obesity-associated gene *MC4R* modifies the ability of the lifestyle intervention to induce weight loss [20], the missense type 2 diabetes-associated variant P446L in *GCKR* modifies the effect of the intensive lifestyle intervention on triglyceride concentrations [21], and a genetic risk score for lipid traits also modifies the response to lifestyle intervention on LDL cholesterol concentrations and small LDL particle number [22]. A combined meta-analysis of the DPP (in prediabetic participants) and the Look AHEAD trial (in participants with established type 2 diabetes) has shown that genotype at the obesity-associated gene *MTIF3* also predicts the degree of weight loss after a lifestyle intervention [23].

With regard to metformin, several genetic variants seem to modify metformin’s ability to reduce diabetes incidence [10]. One that has been shown to have an effect on metformin response in an independent cohort [24] is a polymorphism in the gene *SLC47A1*, which encodes the metformin transporter MATE1. The extent to which genetic loci associated with glycemic response in patients with established type 2 diabetes will exert a similar effect on diabetes prevention awaits more detailed analyses, although preliminary evidence for the top locus unearthed in a genome-wide association study of metformin response [25] indicates that the two traits may not have perfectly overlapping genetic architectures [26]. That is to say, the genetic variants that modify glycemic response to metformin in a person with established diabetes may not necessarily influence metformin’s ability to prevent diabetes and vice versa, in that the two processes may act via related but separate molecular pathways at different stages of disease progression.

## Conclusion

In summary, while type 2 diabetes represents one of the most serious threats to global public health in the 21st century, strategies exist to stem its spread. To rationalize deployment of diabetes prevention strategies in a cost-efficient manner, it may help to stratify the population into groups at highest risk or most likely to benefit. Genetic prediction does not seem to provide much additional information beyond traditional clinical predictors in identifying those at increased risk, with the potential exception of some variants with strong effects that are more prevalent in specific ethnic groups; thus, if included, genetic predictors should always be considered in conjunction with other markers to obtain an overall estimate of risk. Whether the suggestive evidence that genetic predictors may help stratify response to preventive interventions is eventually translated into clinical practice awaits the completion of well-powered clinical trials.

## Abbreviations

DPP: U.S. Diabetes Prevention Program
MODY: maturity-onset diabetes of the young
